# Photobiomodulation as a Potential Regenerative Signal in the Inner Ear: Linking Mitochondrial Redox Signaling to Cell-Fate Regulation

**DOI:** 10.3390/ijms27198438

**Published:** 2026-09-22

**Authors:** Min Young Lee

**Affiliations:** 1 Beckman Laser Institute Korea, Dankook University, Cheonan 31116, Republic of Korea; eyeglass210@gmail.com; 2 Medical Laser Research Center, College of Medicine, Dankook University, Cheonan 31116, Republic of Korea; 3 Department of Otolaryngology-Head & Neck Surgery, College of Medicine, Dankook University, Cheonan 31116, Republic of Korea

**Keywords:** photobiomodulation, inner ear, hair-cell differentiation, neural protection, Wnt, Notch, cell fate

## Abstract

Photobiomodulation (PBM) is a non-thermal therapeutic modality in which low-intensity red or near-infrared light is used to modulate biological activity. Experimental inner-ear studies report preservation of auditory neurons, attenuation of vestibular injury, improved survival of transplanted stem cells, and changes in otic organoid differentiation. These observations raise the possibility that PBM may influence differentiation-associated cellular states in addition to providing cytoprotection. The classical mechanistic model proposes that photons interact with mitochondrial chromophores, particularly cytochrome c oxidase, leading to changes in respiration, ATP, reactive oxygen species (ROS), nitric oxide, calcium, and downstream signaling; however, a universal primary photoreceptor has not been established. In a 2020 mouse embryonic-stem-cell-derived otic-organoid study, PBM was associated with more organoid-like structures, increased hair-cell-associated markers, increased ROS, and lower *Hes5* expression. The RNA-sequencing comparison pooled five embryoid bodies into one sample per condition, limiting biological replication and statistical inference. Accordingly, the *Hes5* result should be regarded as exploratory and does not demonstrate direct Notch inhibition. Studies in other regenerative tissues show that PBM can engage ROS-dependent PI3K/AKT/GSK3β/β-catenin signaling, providing a mechanistic precedent rather than proof for the inner ear. This review therefore presents a testable working model in which PBM may connect mitochondrial and redox responses to Wnt/β-catenin and Notch/HES5 pathways during otic differentiation, while emphasizing that mature hair-cell regeneration, new SGN generation, and hearing restoration have not yet been established.

## 1. Introduction

Sensorineural hearing loss is characterized by irreversible or poorly reversible damage to cochlear hair cells, supporting cells, synaptic structures, and spiral ganglion neurons (SGNs). Because mature mammalian cochlear hair cells have very limited spontaneous regenerative capacity, restoration of hearing after extensive sensory-cell or neuronal loss remains a major challenge [1]. Current clinical approaches, including hearing aids [2] and cochlear implantation, compensate for lost function but do not reconstruct damaged sensory or neural structures. Regenerative strategies therefore include stem-cell transplantation, endogenous progenitor activation, gene therapy, growth-factor delivery, and organoid-based approaches [3,4,5]. Foundational studies have established that ATOH1 is required for hair-cell development and that forced ATOH1 expression can induce hair-cell-like phenotypes [6,7,8,9,10]. Wnt/β-catenin signaling regulates cochlear progenitor proliferation and hair-cell differentiation, whereas Notch/HES signaling helps maintain supporting-cell states and restrict hair-cell fate [11,12,13,14,15,16]. These developmental pathways provide the biological framework for asking whether a physical stimulus such as PBM can modify progenitor state.

PBM uses low-intensity red or near-infrared light to modify cellular physiology and has been associated with mitochondrial, redox, inflammatory, proliferative, and differentiation responses [17,18,19]. Direct inner-ear evidence is nevertheless limited and unevenly distributed. Several of the studies most central to the present hypothesis—including the auditory-neuropathy, vestibular, otic-organoid, and transplantation studies—were produced by the same research group [20,21,22,23,24]. Independent studies provide complementary evidence for cytoprotective or stress-modulating effects in auditory-cell and noise-injury models [25,26], whereas a randomized placebo-controlled human study did not detect significant auditory benefit [27]. This concentration of evidence does not invalidate the findings, but it limits independent replication and should temper mechanistic and translational claims.

Throughout this review, “protection” denotes preservation of existing cells or function after injury; “repair” denotes partial restoration of damaged structures without proof of newly generated, integrated cells; and “regeneration” denotes the production or replacement of lost cells followed by maturation, structural integration, and functional contribution. Under these definitions, the current SGN evidence supports neural protection, not demonstrated neurogenesis or SGN regeneration. The central question is therefore whether PBM may become a component of a regenerative strategy, rather than whether it has already been shown to regenerate the inner ear.

## 2. PBM in the Inner Ear: From Cytoprotection Toward Regenerative Hypotheses

The early inner-ear PBM literature primarily supports tissue protection. In ouabain-induced auditory neuropathy, 808 nm PBM improved ABR thresholds and preserved SGNs, neurofilaments, and postsynaptic puncta [20]. The study did not demonstrate birth of new neurons, lineage conversion, or functional integration of new SGNs; it should therefore be interpreted as neural protection rather than regeneration. In gentamicin-induced vestibular dysfunction, near-infrared PBM improved vestibular function and reduced histological injury [21]. Independent studies likewise reported stress-modulating or protective effects in auditory cells and noise-exposed cochleae [25,26]. Together, these data support a plausible cytoprotective action across sensory and neural compartments, but they do not show replacement of lost inner-ear cells.

The distinction between survival and regeneration is especially important for cell transplantation. In the 2023 secondary-neurodegeneration study, PBM increased the persistence and distribution of transplanted GFP-positive embryonic stem cells, but neither ABR nor eABR recovered and nerve regeneration was not demonstrated [23]. One of three animals in the combined stem-cell-plus-PBM group had a deformed cochlea with widespread GFP-positive cells and absent neurofilament expression in the abnormal sample. A single animal cannot establish toxicity, but the finding is a safety signal that warrants cautious reporting, longer follow-up, lineage tracing, and larger studies. Enhanced survival is necessary for transplantation strategies, but it is insufficient without controlled lineage specification, maturation, synapse formation, and integration into the auditory circuit.

The current evidence therefore supports a transition from studying PBM solely as a cytoprotective intervention toward testing it as a possible adjunct to regeneration. That conceptual shift should not be confused with evidence that PBM itself regenerates hair cells or SGNs. A systematic review identified 17 eligible PBM studies across animal, human, and ex vivo models, with substantial heterogeneity in wavelength, dose, delivery, and endpoints [28]. Standardized dosimetry, independent replication, and functional endpoints are prerequisites for stronger claims (Table 1).

Evidence-level criteria: “Moderate” indicates direct inner-ear observations with biological replication and/or support from independent studies, but incomplete causal or functional validation. “Preliminary” indicates limited direct evidence from a small number of studies or incompletely replicated experiments. “Very limited” indicates indirect, pooled, under-replicated, or association-only evidence. “Not established/Unknown” indicates no direct demonstration. Ratings also consider whether evidence is inner-ear-specific or extrapolated, independent replication, biological replicate number, association versus intervention, and whether endpoints are molecular, structural, or functional.

## 3. PBM and Inner-Ear Organoid Differentiation

The development of inner-ear organoids provides a useful platform for determining whether PBM may modify differentiation-associated cell states rather than simply preserve existing cells. In mouse embryonic-stem-cell-derived embryoid bodies, 630 nm LED PBM was associated with more organoid-like structures and increased expression of otic and hair-cell-associated markers, including *Pax2*, *Pax8*, *Atoh1*, and *Myo7a*. MYO7A-positive cells and FM1-43 uptake were also reported [22]. These endpoints support altered differentiation and the presence of hair-cell-like features, but they do not by themselves establish mature functional hair cells or regeneration in vivo.

The transcriptomic component of the 2020 study should be interpreted more cautiously than the original wording of this review implied. For each condition, five embryoid bodies were pooled to generate the RNA-seq sample, leaving no independent biological replicate at the sequencing level [22]. The observed decrease in *Hes5* is therefore preliminary and hypothesis generating. Because HES5 is a downstream effector of Notch signaling, lower *Hes5* expression is compatible with altered Notch-related transcription, but it does not prove direct inhibition of Notch. Direct evidence would require replicated measurements of NICD generation or localization, RBPJ-dependent transcription, and pathway perturbation or rescue.

The strengths and limitations of the organoid evidence should be considered together: PBM-associated differences were detected across organoid yield, marker expression, ROS, and cellular morphology, but the model remains an early developmental system, the transcriptomic comparison was under-replicated, and independent laboratories have not yet reproduced the cell-fate result. Future studies should predefine biological replicates, analyze individual organoids or independent differentiation batches, and connect molecular changes to electrophysiological maturation.

The effects of PBM on otic stem-cell behavior are not restricted to the 630 nm wavelength. A subsequent study examined PBM at wavelengths above 800 nm in stem cells within otic organoids and the scala media of the cochlea and demonstrated morphological changes in stem cells together with increased formation of otic vesicle-like structures, suggesting that near-infrared PBM can modify cellular behavior within an otic developmental environment [24]. The observation that both visible red and near-infrared wavelengths can influence otic stem-cell behavior raises an important question regarding the relationship between wavelength and molecular mechanism. The biological response to PBM is strongly dependent on wavelength, irradiance, fluence, exposure duration, treatment frequency, tissue depth, and cellular state, and a systematic review of PBM for hearing loss identified 17 eligible studies with considerable variation in treatment parameters [28]. Most animal studies used wavelengths between 808 and 830 nm, whereas inner-ear organoid studies included both 630 nm and wavelengths above 800 nm. Such heterogeneity complicates direct comparison between studies and makes it difficult to determine whether different wavelengths converge on a common molecular pathway or activate distinct biological mechanisms. A major future objective should therefore be to determine whether red and near-infrared PBM generate a common cellular state despite differences in photon absorption, tissue penetration, and intracellular targets.

ATOH1 is a central transcription factor in cochlear hair-cell specification [6,7,8,9,10], and Wnt/β-catenin and Notch signaling regulate the balance among progenitor proliferation, supporting-cell identity, and hair-cell differentiation [11,12,13,14,15,16]. Nevertheless, increased *Atoh1* or *Myo7a* transcript abundance is a differentiation-associated molecular endpoint, not proof of functional regeneration. A mature hair cell must acquire organized stereocilia, mechanotransduction currents, appropriate electrophysiological properties, ribbon synapses, and functional connectivity. FM1-43 uptake in the PBM-treated organoids provides an additional functional clue [22], but ultrastructural, electrophysiological, and synaptic validation remains necessary.

Inner-ear organoids remain an attractive platform for mechanistic PBM research because they permit assessment of differentiation, spatial organization, developmental timing, and interactions among multiple cell populations [29]. PBM could be tested alongside defined Wnt activation and Notch inhibition schedules [11,16], but such combinations should be framed as experimental strategies rather than established protocols. The next step is to determine whether PBM reproducibly changes lineage allocation across independent differentiation batches and whether any increase in hair-cell-like cells is accompanied by maturation, synaptic connectivity, and functional integration.

## 4. Molecular Mechanisms of PBM: Mitochondria, ROS, Wnt, and Notch

A widely discussed PBM model proposes that photons interact with mitochondrial chromophores, particularly cytochrome c oxidase, thereby modifying electron transport, mitochondrial membrane potential, ATP production, ROS generation, and nitric oxide signaling [17,18]. Mitochondria are central to hair-cell bioenergetics and stress responses, and mitochondrial calcium handling can drive ROS production during hair-cell injury [30,31,32]. These observations make mitochondrial signaling biologically plausible in the inner ear, but they do not identify a universal PBM photoreceptor or show that the same mechanism controls otic differentiation. Reviews and isolated-enzyme experiments have questioned whether cytochrome c oxidase alone can account for red or near-infrared responses [33,34]. A contemporary working model should therefore allow multiple photoreceptive, mitochondrial, membrane, ion-channel, and intracellular mechanisms [35] (Table 2).

ROS can act either as mediators of injury or as transient second messengers, depending on magnitude, timing, compartment, and cellular state. The otic-organoid study reported higher ROS after PBM together with altered differentiation markers [22], but did not establish that ROS was necessary for the phenotype. In a hair-follicle stem-cell model, by contrast, ROS scavenging and β-catenin manipulation provided causal evidence linking PBM-induced ROS to PI3K/AKT/GSK3β/β-catenin signaling and hair regeneration [36]. Neural-injury studies have also reported increased neuroprogenitor markers after transcranial PBM [37]. These cross-system findings support experimental hypotheses for the inner ear; they should not be presented as evidence that PBM has already activated the same regenerative pathways in cochlear progenitors.

Wnt/β-catenin signaling is a major regulator of cochlear development, sensory progenitor proliferation, and hair-cell differentiation [12,13,14]. Sequential Wnt activation and Notch inhibition can promote hair-cell production in postnatal cochlear models [16]. Outside the auditory system, PBM has increased Wnt-related readouts in outer-root-sheath cells [38] and produced causal β-catenin-dependent regeneration in hair follicles [36]. These studies provide mechanistic precedents, but direct PBM-induced Wnt/β-catenin activation has not been demonstrated in otic cells. Inner-ear studies should therefore use β-catenin reporters, pathway inhibitors, genetic perturbation, and rescue experiments before assigning Wnt a causal role.

Notch signaling regulates the balance between supporting-cell and hair-cell fates during cochlear development. NICD interacts with RBPJ to induce HES-family transcriptional repressors, whereas Notch inhibition can permit ATOH1-dependent hair-cell differentiation and, in selected injury models, hair-cell regeneration [11,15,16]. The exploratory decrease in *Hes5* observed after PBM is therefore a reasonable basis for a hypothesis [22], but not evidence of direct Notch inhibition. The sequencing design used one pooled sample per condition, and proximal pathway readouts were not measured. Replicated NICD, RBPJ-reporter, and HES5 measurements, followed by gain- and loss-of-function rescue, are required to establish causality.

A testable possibility is that PBM-induced metabolic or redox changes alter the responsiveness of otic progenitors to multiple developmental pathways rather than directly targeting a single pathway. In this model, β-catenin activation and reduced Notch/HES5 activity could converge on ATOH1-associated differentiation. At present, the Wnt branch is extrapolated from other tissues and the Notch branch is supported only by an exploratory downstream transcript. The model should therefore guide experiments, not be interpreted as a demonstrated molecular sequence in the inner ear.

## 5. PBM as a Potential Modulator of Inner-Ear Cell State and the Regenerative Microenvironment

PBM may be most usefully conceptualized as a context-dependent physical modulator of the regenerative microenvironment rather than a single-target pharmacological treatment (Table 3). Stem-cell differentiation depends on growth factors, extracellular matrix, cell–cell interactions, metabolic state, and developmental signaling. PBM could modify cellular responsiveness through mitochondrial, redox, membrane, or ion-channel effects. In mature injured cochlear tissue, these effects may primarily support survival; during an organoid differentiation window, they may alter differentiation-associated cellular states. This context dependence may help explain why similar wavelengths or doses produce different outcomes across models.

Figure 1 summarizes the proposed mechanism while distinguishing direct inner-ear observations from extrapolated or hypothetical steps. PBM may initiate responses through one or more unconfirmed photoreceptive mechanisms, followed by changes in mitochondrial activity and redox mediators. These events may engage Wnt/β-catenin, or Notch/HES5 signaling and thereby alter ATOH1-associated differentiation. The same upstream responses may also support SGN survival. The current data support mitochondrial/redox changes, exploratory otic differentiation associations, and neural protection; they do not yet establish the complete pathway, mature hair-cell regeneration, or new SGN generation.

## 6. From Association to Causality: Current Mechanistic Limitations and Experimental Strategies

The most important limitation of the current PBM literature is the weakness of direct causal evidence. Many studies report changes in markers, but few establish that the marker is required for the biological response. Reduced *Hes5* after PBM does not prove direct Notch inhibition [22], and increased β-catenin in a non-otic model does not establish a cochlear Wnt mechanism [36,38]. The inner-ear evidence base is also concentrated in a small number of studies, several from the same research group [20,21,22,23,24], and the key RNA-seq comparison pooled embryoid bodies without independent sequencing-level biological replicates [22]. Additional limitations include uncertainty regarding the primary photoreceptor [33,34,35], heterogeneous wavelengths and dosimetry [28], short follow-up, small animal cohorts, and reliance on molecular or histological endpoints. Marker-positive cells should not be equated with regenerated functional cells.

The central challenge for the field is to establish a causal chain between photon exposure and regenerative outcome. Future studies should therefore move beyond single time-point measurements and investigate the temporal sequence of molecular events. A particularly informative experiment would simultaneously measure ROS, mitochondrial membrane potential, ATP, NICD, HES5, β-catenin, ATOH1, POU4F3, GFI1, and MYO7A at multiple time points after PBM. If ROS changes precede alterations in NICD or β-catenin and these changes subsequently precede ATOH1 induction and hair-cell differentiation, a temporal hierarchy would be established. However, temporal association alone would not prove causality. Causal evidence would require pathway perturbation. For example, if inhibition of ROS prevented PBM-induced HES5 suppression, ROS would be positioned upstream of Notch-related signaling. If forced NICD expression prevented PBM-induced ATOH1 and MYO7A expression, Notch signaling would be demonstrated as a necessary mediator. Similarly, if inhibition of β-catenin abolished PBM-induced differentiation, Wnt/β-catenin signaling would become a causal component of the response. Such experiments would transform the field from descriptive PBM biology into mechanistically defined regenerative biology.

## 7. Translational Perspectives and Future Applications

The most realistic near-term role of PBM is as an experimental adjunct that may improve the biological environment for regenerative therapies. Potential preclinical applications include supporting transplanted-cell survival, modulating defined differentiation windows, or preserving SGNs as a substrate for electrical stimulation. However, the 2023 transplantation study increased cell persistence without ABR/eABR recovery or demonstrated nerve regeneration, and one animal showed a deformed cochlea with widespread GFP-positive cells [23]. This isolated finding does not prove toxicity, but it argues for dose control, lineage restriction, tumorigenicity surveillance, and longer safety studies before translation.

Clinical plausibility must be separated from clinical evidence. PBM has theoretical advantages as a non-pharmacological, parameter-adjustable intervention, but light penetration, cochlear dose distribution, target specificity, and treatment timing remain unresolved. Human studies are sparse and heterogeneous; a randomized placebo-controlled study found no significant improvement in auditory outcomes [27], and no human study has demonstrated hair-cell or SGN regeneration. Stage-specific PBM protocols—survival, lineage specification, maturation, and integration—should therefore be tested first in adequately powered preclinical models with functional endpoints.

## 8. Conclusions

PBM research in the inner ear has expanded from cytoprotection to exploratory studies of stem-cell behavior and otic organoid differentiation. Current evidence supports preservation of SGNs and auditory neural structures, attenuation of vestibular or cochlear injury in selected models, and improved survival of transplanted cells [20,21,23,25,26]. The SGN findings constitute neural protection, not demonstrated regeneration. In otic organoids, PBM was associated with increased ROS, hair-cell-associated markers, and lower *Hes5* expression [22]. Because the RNA-seq analysis used one pooled sample of five embryoid bodies per condition, the *Hes5* result is preliminary and does not establish direct Notch inhibition. Evidence from hair follicles and other regenerative systems supports ROS-dependent signaling as a mechanistic precedent [36,37,38], but direct Wnt activation by PBM in the inner ear remains unproven. PBM should therefore be considered a potential regenerative signal or adjunct that may alter differentiation-associated cellular states, rather than an established inducer of inner-ear regeneration. The priority is to test causal pathway perturbations, independent biological replication, lineage tracing, mature hair-cell and synaptic endpoints, long-term ABR/eABR recovery, and safety. Until those criteria are met, claims of functional hair-cell regeneration, SGN regeneration, or clinical hearing restoration are not established.

## Figures and Tables

**Figure 1:**
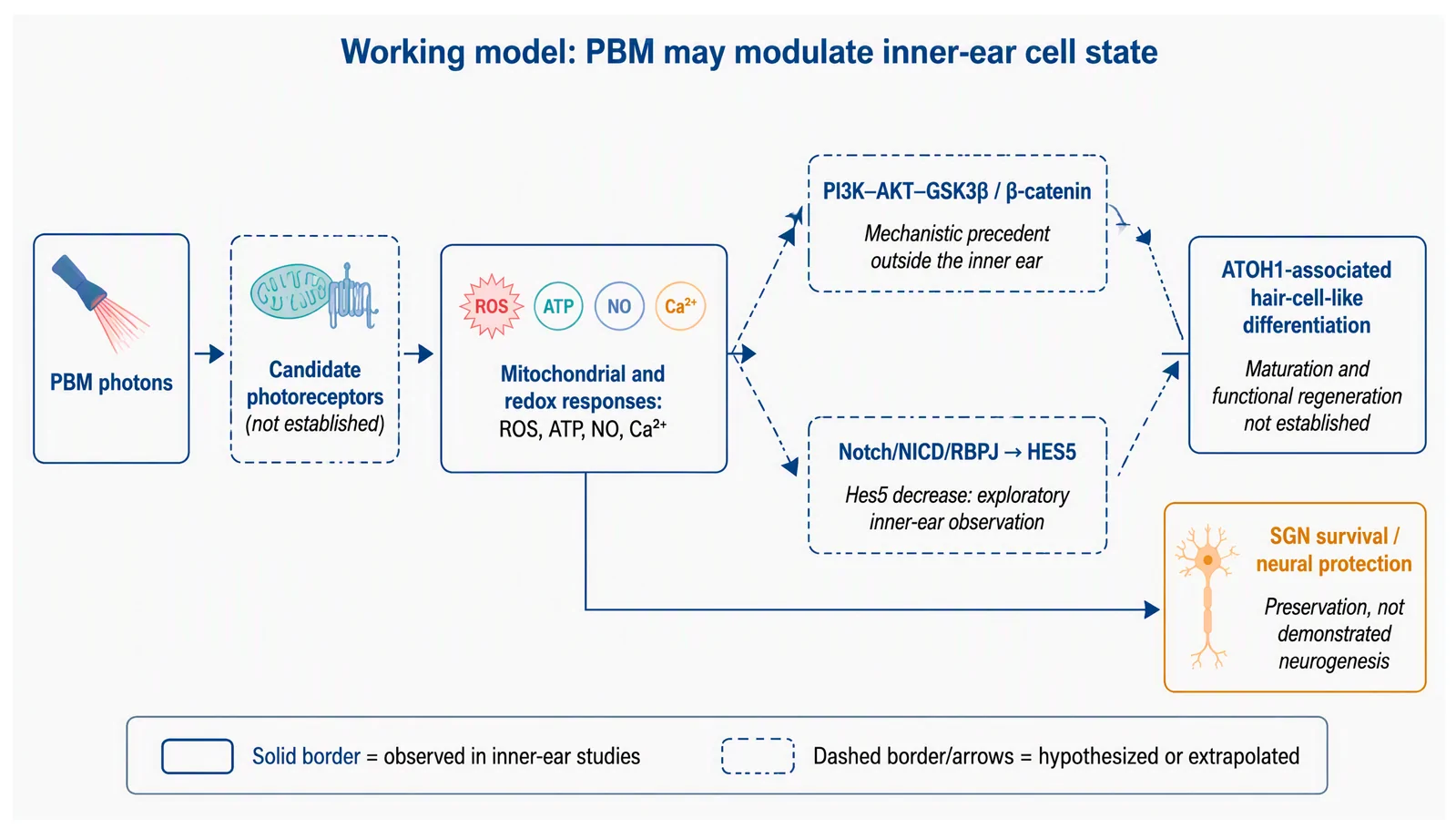
Working model for PBM-associated inner-ear responses. Solid borders and arrows denote observations reported in inner-ear models; dashed elements denote hypotheses or mechanistic extrapolations from other tissues. The decrease in *Hes5* was observed in an exploratory pooled RNA-seq comparison and does not establish direct Notch inhibition. SGN data support neural protection rather than demonstrated neurogenesis. PBM, photobiomodulation; ROS, reactive oxygen species; NO, nitric oxide; SGN, spiral ganglion neuron.

**Table 1:** Representative PBM studies relevant to inner-ear regeneration. Dosimetry is reported as wavelength, irradiance, fluence, and exposure; NR = not reported; N/A = not applicable; calculated fluence values are identified explicitly.

Year	Study	Model	PBM Condition	Major Finding	Mechanistic Significance	PMID
2013	Goodman et al. [27]	Adults with hearing loss; randomized, placebo-controlled	Wavelength: NR; irradiance: NR; fluence: NR; exposure: NR	No significant group differences in audiometry, speech testing, or otoacoustic emissions	Human efficacy not demonstrated	24024040
2016	Bartos et al. [25]	HEI-OC1 auditory cells under oxidative stress	Wavelength: 810 nm; irradiance: 30 mW/cm^2^; fluence: 3 J/cm^2^; exposure: 100 s/session	Reduced inflammatory cytokines and oxidative-stress markers after gentamicin or LPS challenge	Independent cytoprotection/redox evidence	26790619
2016	Lee et al. [20]	Gerbil auditory neuropathy	Wavelength: 808 nm; source power: 200 mW; target irradiance: 909 mW/cm^2^; fluence: 3272 J/cm^2^; exposure: 1 h/day × 7 days; trans-tympanic delivery	ABR improvement and preservation of SGNs, neurofilaments, and postsynaptic puncta	Neural protection	27666974
2016	Tamura et al. [26]	Rat noise induced hearing loss	Wavelength: 808 nm; irradiance: 165 mW/cm^2^; fluence: 297 J/cm^2^/session (calculated); exposure: 30 min/day × 5 days	Accelerated ABR recovery with reduced oxidative-stress and apoptotic markers	Independent cochlear protection	27342816
2017	Lee et al. [21]	Rat vestibulopathy	Wavelength: 830 nm; source power: 200 mW (165 mW at fiber tip); irradiance: NR; fluence: NR; exposure: 30 min/day × 7 days; reported total energy: 297 J	Improved vestibular function and reduced injury	Vestibular protection	28853245
2020	Chang et al. [22]	mESC-derived inner-ear organoids	Wavelength: 630 nm; irradiance: 40 mW/cm^2^; fluence: 30 J/cm^2^/day (90 J/cm^2^ total); exposure: 750 s/day × 3 days	More organoid-like structures and hair-cell-associated markers; exploratory decrease in *Hes5*	Preliminary cell state modulation; Notch hypothesis	32258218
2021	Chang and Lee [24]	Otic organoids and cochlear scala media	Wavelength: 850 nm LED (in vitro) and 808 nm laser (in vivo); irradiance: 40 mW/LED (in vitro); source power: 250 mW (in vivo); fluence: 30 J/cm^2^/day × 3 days (in vitro), 67.5 J/cm^2^ estimated (in vivo); exposure: 750 s/day × 3 days (in vitro), 90 min (in vivo)	Morphological changes in otic stem cells	Preliminary stem cell modulation	33604771
2023	Chang et al. [23]	Cochlear auditory neuropathy plus stem cells	Wavelength: 808 nm; irradiance: 40 mW/cm^2^; fluence: 144 J/cm^2^/session (calculated); exposure: 1 h/day × 5 days	Stem-cell viability increased; no ABR/eABR recovery or nerve regeneration	Survival does not establish regeneration	38020205
2024	Nikookam et al. [28]	Systematic review	Wavelength: multiple; irradiance: N/A; fluence: N/A; exposure: N/A (heterogeneous protocols)	17 studies with substantial parameter and outcome heterogeneity	Need for standardization and independent validation	37885344

**Table 2:** PBM-associated signaling pathways relevant to inner-ear regeneration.

Pathway	Reported PBM Effect	Evidence in Inner Ear	Evidence in Other Regenerative Systems	Potential Role in Inner-Ear Regeneration	Key PMID
Mitochondrial respiration	Altered mitochondrial activity and cellular energetics	Auditory-cell and injury models; differentiation-specific role unresolved	Extensive	Candidate upstream metabolic signal	28070154, 26790619
Cytochrome c oxidase	Proposed photoreceptor	Not established	Controversial	Possible primary photoreceptor	32716711, 27111566
ROS	Increased or modulated ROS	Increased in otic organoids; necessity not tested	Stronger mechanistic evidence	Potential second messenger with dose- and context-dependent effects	32258218, 34019818
Nitric oxide	NO signaling proposed	Limited	Moderate	Mitochondrial and vascular signaling	21356170
PI3K/AKT	Activation	Not directly tested in otic PBM models	Demonstrated	Candidate survival/differentiation pathway	34019818
GSK3β/β-catenin	β-catenin stabilization	Not directly tested in otic PBM models	Strong mechanistic evidence in hair follicles	Candidate Wnt mechanism; extrapolation	34019818
Wnt	Increased Wnt signaling in some non-otic models	Developmentally relevant; PBM activation not demonstrated in the inner ear	Demonstrated in regenerative models	Candidate progenitor activation/differentiation pathway	28944964, 34019818
Notch/NICD	Potential suppression	Lack of direct Wnt/β-catenin measurements	Limited direct PBM evidence	Candidate regulator of hair-cell fate	32258218
*Hes5* transcript	Exploratory decrease	One RNA-seq comparison using five pooled EBs per condition	—	Hypothesis-generating association, not direct Notch inhibition	32258218
ATOH1	Hair-cell-associated expression increased	Organoid marker evidence; mature function not established	—	Hair-cell lineage specification marker	32258218
ERK/MAPK	Activation	Not adequately studied	Demonstrated	Proliferation/differentiation	28944964

**Table 3:** Mechanistic evidence hierarchy and proposed experiments.

Question	Current Evidence	Evidence Level	Recommended Experiment
Does PBM alter mitochondrial metabolism?	Direct auditory-cell data and the broader PBM literature	Moderate	Seahorse analysis, ATP, membrane potential, and action-spectrum studies in otic cells
Is cytochrome c oxidase the primary photoreceptor?	Widely proposed but controversial	Unresolved	CCO manipulation and wavelength/action-spectrum studies
Does PBM increase ROS in otic models?	Increase observed in one organoid study; causal role untested	Preliminary	Replicated time-course and compartment-specific ROS measurements
Is ROS necessary for PBM-associated otic differentiation?	Causal evidence exists in other regenerative tissues, not the inner ear	Very limited	NAC and mitochondrial ROS scavenging with differentiation rescue controls
Does PBM activate Wnt/β-catenin in otic cells?	Mechanistic precedent outside the inner ear	Not established	β-catenin reporter, pathway inhibition, and genetic deletion/rescue
Does PBM inhibit Notch?	Exploratory *Hes5* decrease in a pooled RNA-seq comparison	Very limited	NICD cleavage/localization, RBPJ reporter, and independently replicated *Hes5* assays
Is HES5 required for the PBM-associated phenotype?	Not established	Unknown	HES5 overexpression/knockdown and rescue
Is ATOH1 activation dependent on Notch suppression?	Biologically plausible; not directly tested	Unknown	NICD rescue and ATOH1 loss-of-function experiments
Does PBM produce mature hair cells?	Hair-cell-associated markers and FM1-43 uptake reported	Limited	Stereocilia ultrastructure, mechanotransduction currents, ribbon synapses, and longitudinal function
Does PBM regenerate SGNs?	Survival and structural preservation reported; no new-neuron generation shown	Not established	Birth dating, lineage tracing, neurite targeting, synapse formation, and functional integration
Does PBM restore hearing through regeneration?	No direct demonstration; transplantation study showed no ABR/eABR recovery	Not established	Long-term ABR/eABR linked to lineage-resolved histological and electrophysiological integration

## Data Availability

No new data were created or analyzed in this study. Data sharing is not applicable to this article.
